# Supplementary material for: Mental Health Consequences of the Three Mile Island, Chernobyl, and Fukushima Nuclear Disasters: A Scoping Review
Source: Int J Environ Res Public Health. 2021 Jul 13;18(14):7478. doi: 10.3390/ijerph18147478 (PMC8304648; doi:10.3390/ijerph18147478)
Supplement: Supplementary file 1 [file ijerph-18-07478-s001.zip › ijerph-1252749-Supplementary Material 1.pdf]

International Journal of
Environmental Research and Public Health

MDPI

Supplementary Material S1

Search Formulas

For Pubmed

Whole 2020/07/12
(("Chernobyl" [Title/Abstract] OR “Chornobyl" [Title/Abstract] OR "Chernobyl Nuclear Accident" [MeSH Terms]) OR (“Three Mile" [Title/Abstract]) OR (“Fukushima" [Title/Abstract] OR “Fukushima Nuclear Accident" [MeSH Terms])) AND ((((((((((((((((("Mental Disorders" [MeSH Terms] OR "Mental Health" [MeSH Terms]) OR "Psychological Phenomena" [MeSH Terms]) OR "Behavioral Disciplines and Activities" [MeSH Terms]) OR "Behavior and Behavior Mechanisms" [MeSH Terms]) OR "Quality of Life" [MeSH Terms]) OR "Psychophysiologic Disorders" [MeSH Terms]) OR "Stress Disorders, Post-Traumatic" [MeSH Terms]) OR "Mental Health" [Title/Abstract]) OR "Mental Disorders" [Title/Abstract]) OR "depressi*" [Title/Abstract]) OR "anxiety" [Title/Abstract]) OR "posttraumatic" [Title/Abstract]) OR "post-traumatic" [Title/Abstract]) OR “traumatic" [Title/Abstract]) OR "distress" [Title/Abstract]) OR "psychological" [Title/Abstract]) OR "alcohol" [Title/Abstract])

医中誌
((スリーマイル/AL) or (((チェルノブイリ原発事故/TH or チェルノブイリ原発事故/AL)) and (PT=原著論文)) or (((福島原発事故/TH or 福島原発事故/AL)) and (PT=原著論文))) and (((うつ/AL or (心的外傷/TH or トラウマ/AL) or (ストレス障害-心的外傷後/TH or PTSD/AL) or (不安/TH or 不安/AL) or メンタル/AL or 心理/AL or (Alcohols/TH or アルコール/AL)) and (PT=原著論文)) or (((精神保健/TH or 精神保健/AL) or (精神疾患/TH or 精神疾患/AL) or (心理現象/TH or 心理現象/AL) or (行動訓練と活動/TH or 行動訓練と活動/AL) or (行動と行動メカニズム/TH or 行動と行動メカニズム/AL) or (生活の質/TH or 生活の質/AL) or (心身症/TH or 心身症/AL)) and (PT=原著論文)))

ProQuest(PsycARTICLES and PTSDPub)
("Chernobyl*" OR "chernobyl*" OR "Chornobyl*" OR "chornobyl*") OR "Three Mile" OR (("Fukushima" AND "nuclear") OR ("Fukushima" AND "disaster") OR ("Fukushima" AND "accident"))
