# Supplementary material for: Mental Health Consequences of the Three Mile Island, Chernobyl, and Fukushima Nuclear Disasters: A Scoping Review
Source: Int J Environ Res Public Health. 2021 Jul 13;18(14):7478. doi: 10.3390/ijerph18147478 (PMC8304648; doi:10.3390/ijerph18147478)
Supplement: Supplementary file 1 [file ijerph-18-07478-s001.zip › ijerph-1252749-Supplementary Material 2.pdf]

International Journal of *Environmental Research and Public Health*

MDPI

Supplementary Material 2

Table S1. The studies included in this review.

First author, year [ref.]	Survey period	Study subjects	Control group	Measures	Main findings	Risk factors	Protective factors
**Category A:** Including results of an identical symptom scale changes over time; The articles are arranged in chronological order after each event.							
TMI							
Bromet 1982 [14]	(0–3 mos., 9–10 mos., 12–13 mos.)	Patients at community mental health centers serving the TMI area, *n* = 151; 25% of the patients were diagnosed schizophrenia.	Patients from the community mental health center, the comparison site where other nuclear reactors are located, *n* = 64; 20% of the patients were diagnosed schizophrenia	Anxiety or depression episode (0–3 mos.); SCL-90 (9–10 mos., 12–13 mos.)	At 0–3 mos. after the accident, around 40 % had anxiety or depression episodes. The mean scores of the global severity index of the SCL–90 were 1.01 (SD = 0.74) at 9–10 mos. after the accident and 0.88 (SD = 0.72) at 12–13 mos. after the accident. No significant differences were found between the TMI and Beaver Valley samples across the surveys.	Viewing TMI as currently dangerous	Older and better educated patients were less symptomatic on the SCL-90
Dohrenwend 1981 [15]	Apr 1979, May 1979, Jul–Sep 1979 (1, 2, 3–5 mos.)	General population, within 32 km radius of TMI n= 50, 54, 380	n.a.	Demoralization Score	Average of the demoralization score was around 20 points in April; around 15 points in May and July–August	Living within 8 km of the plant	
Goldsteen 1989 [16]	Sep 1979 (5 mos., time 1), Aug 1982 (3 yrs, time 2)	391 in time 1; 254 in time 2 (among the original respondents); residents within 16 km of the TMI	n.a.	Distress (original multi-item scale)	Distress levels at time 1 was 23.6 (SD 18.4); at time 2 was 29.7 (SD 21.0).	At time 1, trust in TMI-related authorities, perceived danger, perceived harm to health. At time 2, only perceived harm to health	
Goldsteen 1982 [17]	Oct 1979 and Mar 1980 (7 and 12 mos.)	100 residents within 2.4 km of the TMI	n.a.	Demoralization	Mean demoralization score at time 1 was 25.7 at time 1; 26.6 at time 2.		
Bromet 1982 [18]	Time 1: Dec 1979 and Jan 1980 (8–9 mos.) Time 2 (follow up): March 1980 to May 1980 (12–14 mos.)	Time 1: 328 mothers of preschool children living within 10 mi of the TMI nuclear power plant, 189 workers at the plant, and 177 mental health system clients Time 2: 312 mothers, 161 workers and 151 clients	Time 1: 312 mothers, 161 workers, 151 clients in another city (Beaver County). Time 2: 124 mothers, 124 worker, 64 clients in another city	SCL-90	For all categories (i.e., TMI mothers, workers, and mental health system clients), study participants were less symptomatic at Time 2 than at Time 1. TMI mothers had an excess risk of experiencing anxiety and depression during the year after the accident. The risk ratio for new cases of anxiety/depression post-TMI was 3.4 compared with 1.8 for all cases occurring during that one-year period. No differences were observed between the TMI workers and controls. The rates of anxiety/depression of workers were considerably lower than those reported for the mothers. No differences between two patient groups on the SCL-90 GSI 9 and 12 mos. after the accident.	Mothers: psychiatric history before the accident, living within 8 km of the plant, less adequate social support	For both TMI mothers and workers, reporting greater social support from their network of friends and relatives at the anniversary.
Dew 1987 [19]	9, 12, 30, 42 mos.; 54 mos. only for the control group	257 women lived within 16 km of the TMI, who delivered a child in the period between January 1978 and March 1979 (the month of the TMI accident)	104 women lived near the Beaver Valley-Shipping port (BV-S) nuclear plants, who delivered a child in the period between January 1978	SCL-90	TMI women averaged higher symptom levels than BV-S women across all four interviews, $F(1, 356) = 4.58$, $p < 0.04$. Site X Time interaction showed that the TMI accident intensified TMI women's symptom levels during the two initial interviews relative to BV-S women, whereas unemployment (anticipated the	Women from either site with a prior history of psychiatric disorder had substantially higher symptom levels than those without such a history, $F(1, 356) = 17.43$, $p < 0.001$.	

			and March 1979 (the month of the TMI accident)		third interview and occurring thereafter) intensified the BV-S women's symptoms relative to the TMI sample at the two later interviews. This contrast was significant, F (1, 1071) = 8.44, $p < 0.01$	
Bromet 1990 [20]	9, 12, 30, 42 mos. and at restart (81 mos.)	At the time of 42 mos. after the accident: 385 mothers, 176 workers	182 mothers and 149 workers near an undamaged plant; 174 mothers and 153 workers near two coal-fired plants	SCL-90	Mothers of young children remained a vulnerable group. In contrast, workers employed at TMI experienced an initial surge of distress that was short-lived.	
Dew 1993 [21]	9, 12, 30, 42, 81, 120 mos.	110 mothers within 10 miles of TMI	n.a.	SCL-90	Cluster analysis revealed 2 groups: The largest group (n=71) consisted of individuals with uniformly low levels; the second group (n=38) included individuals whose symptom levels were significantly higher at all time points. Their distress levels dropped during the ensuing several yrs, and then climbed again at the occasions of the restart of the undamaged reactor 81 mos. after the accident and the 10th anniversary of the accident.	Higher income, perceiving the situation to be more dangerous, and evacuating at the time of the accident
Baum 1993 [22]	From 1980 to 1984: every 6 mos., from 1985 to 1990: once a year	54 people living within 8 km of the TMI (in 1980; an additional group of TMI residents were added in 1982)	32 control subjects living about 128 km from TMI (an additional group of control area residents was added in 1982)	IES	Higher symptoms of TMI group than the control group held up for 6 yrs.	More intrusive thoughts about the accident, or its aftermath
Davidson 1991 [23]	Summer and Nov of 1985 (6 yrs, 2 mos. before and I month after restart of the undamaged nuclear reactor at TMI)	38 people within 8 km radius of TMI	18 people living in a city some 129 km from TMI	SCL-90R	TMI subjects reported more somatic complaints at two timepoints. The restart did not heighten stress responding in the TMI group.	
Prince-Embury 1995 [24]	1985, June 1989 (6 and 10 yrs)	Random sample of 214 adult residents in households (122 females and 92 males) near the TMI, $n = 117$ in 1985 and $n = 64$ in 1989	n.a.	SCL-90R	A lowering of psychological symptoms between 1985 (GSI mean 0.61 (SD 0.61)) and 1989 (GSI mean 0.34 (SD 0.46)) (t = 4.53, $p < 0.001$) in spite of increased lack of control, less faith in experts and increased fear of developing cancer.	
**Chernobyl**						
Koscheyev 1993 [25]	Time 1: Jul 1986<br>Time 2: Sep–Oct 1986<br>Time 3: Mar–Apr 1987<br>Time 4: Nov–Dec 1987	Time 1: 55 chief operators<br>Time 2: $n = 111$<br>Time 3: $n = 106$<br>Time 4: $n = 97$	110 chief operators at the Ignalina nuclear power station, who were assessed in Apr 1988	MMPI	Significant group differences occurred on all scales except Scales 5 (Masculinity-Femininity), 7 (Psychasthenia), and O (Social Introversion). Post-hoc comparisons demonstrated significant increases in mean scores at some point over time in the Chernobyl groups on Scales 1 (Hypochondriasis), 2 (Depression), 3 (Hysteria), 4 (Psychopathic Deviate), and 8 (Schizophrenia), and a significant decrease on Scale 6 (Paranoia). MMPI mean scale scores at Time 1 were not significantly different from the control group except for Scales 2 and 9 (Hypomania). Comparisons of the Time 2 through Time 4 Chernobyl assessment	

					intervals showed significant increases in mean scores on Scales 1 and 2. 18.4% at Time 1, 20.5% at Time 2, 29.2% at Time 3, 33% at Time 4 of workers showed elevated clinical scale scores. In the control groups, 10% workers showed elevated clinical scale scores.	
Cwikel 1998 [26]	Time 1: 1993–1995 (7–9 yrs) Time 2: 1995–1996 (9–10 yrs)	Time 1: 121 more exposed by Chernobyl, 253 less exposed immigrants from CIS to Israel, TIme 2: 73.3% respondents of Wave 1	Time 1: 334 no exposed immigrants, TIme 2: 73.3% respondents of Wave 1	IES; CES-D; SCL-90	For depression, a significant decrease was found between the first and second wave of interviews (significant main effect of time, F (1, 491) = 122.63, $p < 0.001$). For somatization, the young age groups (18 to 39) decreased their somatization scores, while the older age groups did not. For PTSD, between the first and second interviews, there was a significant decrease over time in the level of PTSD reported (F (2, 478) = 128.9, $p = 0.0001$), but the significant exposure effect remained.	Depression scores significantly increased with age.
Cwikel 1997 [27]	Time 1: 1994 (8 yrs) Time 2: 1996 (10 yrs)	Time 1: 374 immigrants to Israel from the exposed areas Time 2: 304 immigrants	Time 1: 334 immigrants from unexposed areas Time 2: 216 immigrants	IES; CES-D; SCL-90	The results obtained in the first wave conducted 8 yrs after the accident showed that psychological symptoms were significantly higher in exposed respondents than in the comparison group. During the second wave (10 yrs after the accident) we observed a decline in the prevalence of PTSD and related distress except for somatization, which remained at the same level. Mean (SD)scores for PTSD on time 1:8.9(10.4); time 2:3.7(7.0), $p < 0.0001$.A significant interaction effect for exposure x time (F (3,482) = 7.85, $p < 0.0001$). The source of this interaction is from the relatively greater decrease in PTSD scores for the comparison group relative to the decrease in the exposed groups. The clean-up workers are the most depressed at time1, but this disappears by time2 because there is no significant exposure effect at this point. For anxiety scores, a significant effect for exposure was found at both time points, particularly among members of the more exposed group, and a significant decrease over time ($p < 0.01$ and p<0.001 for exposure and time, respectively). For somatization, there is a significant exposure effect but no change over time ($p < 0.0001$).	
Rahu 2014 [28]	From 2004 to 2012	3680 Estonian Chernobyl cleanup workers through the Population Registry and Health Insurance Fund database.	7631 men (population sample) through the Population Registry and Health Insurance Fund database	Three-digit ICD-10 code	No increase in morbidity for stress reactions, depression, headaches, or sleep disorders was detected.	
			**Fukushima**			

Ikeda 2019 [29]	May–June 2011, 2012, 2013, 2014	1,403 full-time TEPCO employees of the Fukushima nuclear power plant (Daiichi and Daini) who completed a questionnaire concerning their disaster-related experiences in 2011 and provided at least one assessment of insomnia-related symptoms between 2011 and 2014	n.a.	The Athens Insomnia Scale (AIS)	A significant decreasing trend over time for the AIS score. The means score of AIS were 6.5 in 2011; 5.0 in 2012; 5.6 in 2013; 5.8 in 2014	Most types of traumatic exposure in a time-independent way. The effect of social discrimination/slurs on insomnia turned out to be constant over time (OR = 1.82; 95% CI = 1.24 to 2.67) after controlling for PTSR status in the multivariate-adjusted model.	
Kato 2017 [30]	Jan 2011 (2 mos. before the disaster), July 2011, May 2013 (4, 26 mos.)	94 Fukushima Medical University students	n.a.	The Japanese version of the Perceived Stress Scale (JPSS)	Mean JPSS score at baseline was 31.0 (SD 7.1). Mean changes in JPSS from baseline to 4-month follow-up was +0.2.		
Ikeda 2017 [31]	May–June 2011 (2–3 mos.), May–June 2012 (14–15 mos.), November 2013 (32 mos.), November 2014 (44 mos.)	1417 TEPCO workers in 2011; 1101 workers in 2012; 834 workers in 2013; 775 workers in 2014	n.a.	K6; IES-R	A significant decreasing trend over time for the K6 and PTSR-score.; The mean score of K6 were 6.35 in 2011; 4.01 in 2012; 4.88 in 2013; 4.97 in 2014. The mean score of IES-R were 17.0 in 2011; 11.1 in 2012; 13.1 in 2013; 12.2 in 2014.	Experience of life-threatening danger; Discrimination/slurs (For IES-R)	
Sawa 2013 [32]	Aug 2011 and Feb 2012 (Fukushima group); not known (by Jan 2012) and Feb 2012 (control group)	32 adult caregivers of residents with intellectual disabilities in Fukushima Prefecture	41 adult caregivers of residents with intellectual disabilities in Chiba Prefecture	GHQ-12	The mean score of GHQ-12 at time 1 was 6.28 and at time 2 was 6.75 (no significant differences between time 1 and time 2).		
Oe 2017 [33]	Jan 2012, Jan 2013, Feb 2014 (10, 22, 35 mos.)	7757 participants who responded all three assessments of the evacuation zone	n.a.	PCL-S	The mean score of PCL-S declined slightly from time 1 to time 3. 35.4 (SD 15.4) in2012; 33.6 (14.7) in 2013; 32.8 (14.5) in 2014. The most common pattern of PTSD symptoms was the continuous low symptoms (54.9%), whereas a substantial portion of the study population (8.1%) showed chronic dysfunction.	Older age and poor living circumstances in 2013 showed significant negative effects.	Laughter showed significant odds ratio in the Recovered group and the Resistant group
Oe 2016 [34]	Jan 2012, Jan 2013, Feb 2014 (10, 22, 35 mos.)	12,371 residents	n.a.	K6	The mean scores on the K6 were 7.10 (SD 5.92) in 2012; 6.50 (SD 5.68) in 2013; 5.97 (SD 5.44) in 2014. Using group-based trajectory modelling, we identified four trajectory patterns distinguished by the levels of psychological distress, which gradually improved over time in all trajectories.	Subjective sleep insufficiency, problem drinking, poor social support, and perception of radiation risk 3 yrs after the accident were associated with the severity of psychological distress, according to the multivariate analysis.	
Oe 2018 [35]	Jan 2012, Jan 2013, Feb 2014 (10, 22, 35 mos.)	8282 children born after 2 April 1998 and before 1 April 2004 who were elementary school students (i.e., in the first to the sixth grade) on 11 March 2011	n.a.	The emotional symptoms subscale and the peer relationship problems subscale of SDQ	The mean score for emotional symptoms were 2.48 (SD 2.38) in 2012; 2.08 (2.20) in 2013; 1.92 (SD 2.11) in 2014. The mean score for peer relationship problems were 2.13 (1.83) in 2012; 2.06 (1.77) in 2013; 2.07 (1.81) in 2014. Four patterns for emotional symptoms and three patterns	For emotional symptoms, female gender, experience of tsunami and nuclear plant accident, out-of-prefecture evacuees, and insufficient physical activity were associated with the very	

					for peer relationship problems, using group-based trajectory modelling	severe trajectory. In contrast, for peer relationship problems, male gender, experience of nuclear plant accident, and insufficient physical activity were associated with the very severe trajectory.
Oe 2019 [36]	Jan 2012, Jan 2013, Feb 2014 (10, 22, 35 mos.)	The caregivers of 2,616 children in the first–sixth grades of elementary school, who lived in one of the 13 municipalities that were the target areas of the Mental Health and Lifestyle Survey	n.a.	A question on children's victimization from SDQ	Across 3 yrs, around 80% of caregivers responded "not true," 15% responded "somewhat true," and 5% responded "certainly true" in response to a question about bullying victimization of their children.	Being male was significantly associated with the parental recognition of bullying victimization at time 1 and time 3. At time 1, experiencing the nuclear plant explosion was significantly associated with parental recognition of bullying victimization. Age at time 3 was negatively associated with parental recognition of bullying victimization.
Fukasawa 2020 [37]	Time 1: February to April 2016 (5 yrs) Time 2: November 2018 to January 2019 (8 yrs)	927 respondents who were randomly sampled from non-evacuation zone in Fukushima Prefecture	n.a.	K6; PCL-S	The proportions of the respondents with K6 score of 13 and more was 5.0% at time 1 and 5.9% at time 2. The proportions of the respondents with PCL-S6 score of 17 and more was 3.5% at time 1 and 3.9% at time 2.	Distrust in the government at baseline was significantly associated with posttraumatic stress symptoms at follow-up, albeit controlling for baseline symptoms.
**Category B:** Articles without results of an identical symptom scale change over time or articles that use the same results as those published in Category A; The articles are arranged in chronological order after the event.						
**Chernobyl**						
Bromet 2011 [38]	Time 1:1997 (11 yrs), Time 2: 2005–2006 (19 yrs)	Time 1: 300 evacuees, 10–12-year-old children in Kyiv who were in utero or infants at the time of the disaster and their mothers Time 2: 265 evacuee adolescents who was in utero or up to 15 mos. old at the time of the accident	Time 1: 300 classmates and mothers Time 2: 261 classmate controls, and 327 population-based controls	The mothers' risk perceptions in 1997 and 2005–2006; Adolescent-reported Chernobyl perceptions in 2005–2006; 12-month MDD/GAD diagnosis in 2005–2006; Depression/anxiety symptoms in 2005–2006.	Mothers' Chornobyl perceptions in 1997 were significantly associated with their own and with their children's perceptions at age 19. However, these reports were not directly associated with MDD/GAD or depression/anxiety symptoms.	
Guey 2008 [39]	Time 1: 1997 Time 2: 2005–6	Six hundred child-mother dyads (300 evacuees and 300 classmate controls) were initially assessed in 1997 when the children were 11 yrs old and followed up in 2005–6 when they were 19 yrs old.	A population control group (304 mothers and 327 children) was added in 2005–6.	Children's Somatization Inventory; the Stony Brook Child Symptom Inventory; SCL-90 for mother;	Mother's participation was also predicted by initial concerns about her child's health, greater psychological distress, and Chornobyl risk perceptions. In 2005–6, clinic attendance was also associated with the young adults' risk perceptions, depression or generalized anxiety disorder, lower standard of living, and female gender	

				Mother's Chernobyl risk perceptions; CIDI			
				**Fukushima**			
Kuroda 2017 [40]	May 2010 (10 mos. before the accident), May 2013 (26 mos.)	438 elderly persons (aged ≥65 yrs) living in Iitate village who had no depression tendency in 2010. Iitate village was in the evacuation zone.	n.a.	Basic Checklist (BCL)	Of the 438 respondents in the second survey, 163 (37.2%) showed depression tendency.	PDT (a presence of depression tendency) risk was significantly increased by female gender, age, history of diabetes and cognitive disorder.	Increased IADL (instrumental activities of daily living); Engagement in social activities decreased PDT risk in rental accommodation.
Komuro 2019 [41]	Time 1: May to June 2011 (2–3 mos.) Time 2: Nov 2013 (32 mos.)	985 TEPCO workers who had continued to work at either Daiichi or Daini	n.a.	IES-R; questions about alcohol and tobacco use in Wave 2 (increase, decrease, no change, never)	see risk factors	Increased alcohol use was associated with age of 29 yrs or less [aRR (95% confidence interval (95% CI): 1.26 (1.01 to 1.57)], major property loss [1.25 (95% CI 1.02 to 1.55)], and high posttraumatic stress responses (PTSRs) [1.34 (95% CI 1.08 to 1.67)] at Time 1. Increased tobacco use was associated with age of 29 yrs or less [1.46 (95% CI 1.12 to 1.90)] and high PTSR [1.62 (95% CI 1.25 to 2.10)] at Time 1 ($p < 0.05$).	
Tanisho 2016 [42]	Time 1: May–June 2011 (2–3 mos.), Time 2: May–June 2012 (14–15 mos.)	968 (Daiichi, $n = 571$; Daini, n=397) TEPCO workers	n.a.	Discrimination/slurs; experience of life-threatening danger; IES-R; K6; the Japanese version of Peritraumatic Distress Inventory (PDI)	Higher K6 score at time 2 was predicted by higher K6 score at time 1 ($\beta = 0.491$, $p < 0.001$) and discrimination/slurs experiences at time 1 ($\beta = 0.065$, $p = 0.025$, adjusted R2 = 0.24). Higher IES-R score at time 2 was predicted with higher IES-R score at time 1 ($\beta = 0.548$, $p < 0.001$), higher age ($\beta = 0.085$, $p = 0.005$), and discrimination/slurs experiences at time 1 ($\beta = 0.079$, $p = 0.003$, adjusted R2 = 0.36). PDI score at time 1 was not significantly associated with either K6 score or IES-R score at time 2.		
Murakami 2018 [43]	Jan 2012, Jan 2013	34,312 participants who aged 20 and older as of March 2011 and living in one of the 13 municipalities that were the target area of the Mental Health and Lifestyle Survey.	n.a.	Frequency of laughter; risk perception (immediate risk; delayed risk; genetic risk); K6 (as mental health distress) radiation risk perception as an indicator of radiation-related anxiety and the frequency of	Perceived genetic risk in 2011 had a significant inverse association with the frequency of laughter. Lowering of radiation risk perception was significantly associated with the frequency of laughter in the absence of covariates of mental health distress, but not in their presence. Lowering of radiation risk perception was also significantly associated with low mental health distress.		

				laughter as an indicator of well-being.			
Miura 2017 [44]	Jan 2012 and Jan 2013 (10, 22 mos.)	36,056 adult (aged ≥15 yrs on 11 March 2011) participants of the evacuation zone	n.a.	K6; PCL-S; Perceived radiation risk	Psychological distress over the 2-year period was categorized into the following four groups: chronic, recovered, resistant, or worsened. Most participants (80.3%) were resistant to the disaster. A positive association was found between the radiation risk perception regarding immediate effects and the worsened group in women.	Baseline post-traumatic stress disorder (PTSD) or a history of psychiatric disease predicted being in the chronic or worsened group in mid-term course.	
Suzuki 2018 [45]	Jan 2012, Jan 2013, Feb 2014 (10, 22, 35 mos.)	27,744 participants who were at least 15 yrs old as of the onset of the disaster and living in one of the 13 municipalities that were the target area. (29.8% of all respondents over the three survey yrs)	n.a.	Risk perception (delayed effect: genetic effect); PCL-S	The two-cluster models best fit the data for both the delayed and genetic effects. Many participants showed consistently high or low-risk perceptions over all three study yrs (2011–2013) (for delayed effect: 59% and 41% of participants were in the low and high-risk perception groups, respectively; for genetic effect: 47% and 53%, respectively).	Stronger traumatic reactions (> = 50 on the PTSD Checklist–Specific) significantly affected the odds of being in the high-risk perception group for the delayed and genetic effects, with the associations being strongest soon after the disaster.	
Fukasawa 2020 [46]	Time 1: Feb to Apr 2016 (5 yrs), Time2: Feb to Apr 2016 (7 yrs)	2,037 residents of 49 of the 59 municipalities of Fukushima prefecture, excluding the restricted areas close to the nuclear power plant	n.a.	K6; the six-item abbreviated version of PCL-S; radiation risk perceptions; LSNS-6	(see risk factors)	Higher risk perceptions of radiation exposure in the first survey predicted later posttraumatic stress symptoms but not psychological distress after controlling for baseline symptoms or distress.	
Yamanouchi 2018 [47]	Oct 2012 (19 mos.), Oct 2014 43 mos.)	485 long-term evacuees temporarily living in non-evacuation area of Fukushima Prefecture	n.a.	K6	(see risk factors and protective factors)	Factors associated with exacerbation of poor mental health were the stress factors “Uncertainty about future” ($p = 0.048$) and “Loss of purpose in life” ($p = 0.023$).	The stress relief methods “Accepting myself” (odds ratio (OR): 2.15, 95% confidence interval (CI): 1.02–4.51) and “Interactions with others” (OR: 3.34, 95% CI: 1.43–7.79).
**Category C:** Intervention study							
**Fukushima**							
Imamura 2016 [48]	Aug 2014 (baseline), Sep 2014, Nov 2014	18 mothers who have one or more children of preschool age and were living in Fukushima city or a neighboring municipality, were assigned to intervention	19 mothers (same inclusion and exclusion criteria) were assigned to control	K6; the Brief Job Stress Questionnaire (BJSQ)	The BA program showed a marginally significant intervention effect on psychological distress ($p = 0.051$) and physical symptoms ($p = 0.07$) at 1-month follow-up, while the effect became smaller at 3-month follow-up. The effect sizes at 1-month were medium to large (−0.72 and −0.56, respectively).		
